# Cultural Authority and Food Choices: How Intergenerational Power Shapes Maternal Diet in Rural South Africa

**DOI:** 10.1016/j.cdnut.2026.109542

**Published:** 2026-09-28

**Authors:** Praveenkumar Aivalli, Livhuwani Muthelo, Mxolisi Welcome Ngwenya, Frank Adusei-Mensah, Mabitsela Hezekiel Mphasha, Samuel Akinseinde, Joke-Folu Badejo, Fionnuala M McAuliffe, Catherine M Phillips

**Affiliations:** 1 UCD School of Public Health, Physiotherapy and Sports Science, University College Dublin, Belfield, Dublin 4, Ireland; 2 Department of Nursing Science, University of Limpopo, Mankweng 0727, South Africa; 3 Institute of Public Health and Clinical Nutrition, School of Medicine, Faculty of Health Sciences, University of Eastern Finland, Kuopio, Finland; 4 Department of Public Health and Health Systems, University of Limpopo, Mankweng 0727, South Africa; 5 UCD Perinatal Research Centre, School of Medicine, University College Dublin, National Maternity Hospital, Dublin, Ireland

**Keywords:** maternal nutrition, intergenerational power, cultural authority, grandmother influence, South Africa, qualitative study

## Abstract

**Background:**

Maternal malnutrition remains a persistent public health challenge in South Africa, affecting both maternal and child health outcomes. Dietary practices during pregnancy and lactation are shaped by complex social dynamics that extend beyond individual choice.

**Objectives:**

This study explored maternal dietary practices during pregnancy, defined as the foods women reported consuming, avoiding, substituting, or concealing, and examined how cultural norms and intergenerational power relations influenced these practices within the context of routine antenatal nutrition counseling.

**Methods:**

We conducted a qualitative study in Capricorn district of Limpopo province (March–October 2025). Data comprised focus group discussions (*n* = 22) and individual interviews (*n* = 13) with 150 participants including pregnant women, grandmothers, fathers, community leaders, community health workers, mother mentors (lay peer counselors attached to clinics who support pregnant women and new mothers), and healthcare providers. Data were analyzed using reflexive thematic analysis. Interpretation drew on the Social Ecological Model and Bourdieu’s Theory of Practice.

**Results:**

Four interconnected themes explained dietary behavior. First, elder women, especially grandmothers and mothers-in-law, held decisive authority based on experiential knowledge framed as care, which often was accepted over healthcare advice. Second, pregnant women balanced competing guidance, using respectful deference in public and selective adherence in private. Third, poverty, distance, and poor infrastructure made clinical advice largely unattainable. Fourth, digital messaging and peer networks provided supplementary information that supported dialog but did not displace elder authority.

**Conclusions:**

Reported maternal dietary practice is shaped by intergenerational power dynamics and by resource constraints rather than by nutritional knowledge alone. Grandmothers enforce cultural dietary restrictions that conflict with clinical guidance, whereas pregnant women use public deference and private adaptation to navigate competing advice. Nutrition interventions must engage elders as partners and employ culturally respectful harm-reduction strategies.

## Introduction

Maternal nutrition during pregnancy critically determines maternal and child health outcomes [[1], [2], [3], [4]]. Inadequate nutrition increases risks of low birth weight, preterm birth, fetal growth restriction, and maternal and neonatal morbidity and mortality [[5], [6], [7]]. Globally, an estimated 20 million infants are born with low birth weight annually, with poor maternal nutrition a major contributor in low-income and middle-income countries [8]. Maternal malnutrition drives intergenerational transmission of undernutrition, with children of malnourished mothers facing elevated risks of stunting, impaired neurodevelopment, and chronic disease [[8], [9], [10]]. In the mother, micronutrient deficiencies contribute to anemia and to obstetric complications, whereas in the infant they are associated with impaired growth, poorer neurodevelopment, and greater susceptibility to infection [11,12].

In South Africa, despite sustained investment in maternal health infrastructure, nutritional vulnerability among pregnant women remains widespread [[13], [14], [15], [16]]. The country faces a triple burden of malnutrition: persistent undernutrition, rising overweight and obesity, and ongoing micronutrient deficiencies [[17], [18], [19]]. National maternal mortality is estimated at 88–140 per 100,000 live births, with marked regional disparities [20]. In rural areas, challenges are compounded by food insecurity, limited dietary diversity, weaker market access, and inadequate infrastructure [21]. These challenges are not experienced uniformly across South Africa but are shaped by the social, economic and food environments in which pregnant women live. Rural and urban settings therefore differ in how food is accessed and dietary decisions are made.

In urban settings, pregnant women depend almost entirely on cash purchases, contend with precarious incomes and informal settlement living, and have ready access to inexpensive energy-dense foods, a configuration associated more strongly with overweight and micronutrient deficiency than with dietary shortfall alone [14,22]. In rural, kin-dense settings, family members play an important role in deciding what pregnant women eat. Food is usually bought, prepared, and shared within the extended family. Older family members often have an important influence on these decisions. Pregnant women may have limited control over what food is available and what they eat. This makes rural settings important for understanding how family relationships and older family members influence maternal diet.

Research documents the influence of cultural beliefs and food taboos on maternal diets in sub-Saharan Africa [16,23], and evidence shows that intergenerational power dynamics, particularly the influence of grandmothers, play decisive roles in maternal nutrition decision making [24,25]. Poverty, gender roles, and family hierarchies can shape dietary practices, including the greater influence of elders over clinical advice [16,26]. Important gaps nonetheless remain. Few studies examine how authority is enacted in daily food decisions, how pregnant women negotiate tensions between clinical and traditional advice, and what forms of agency are possible within household power structures. Existing interventions often assume that nutrition education alone will translate into improved dietary practice, overlooking the relational, cultural, and structural contexts in which food decisions are made [16,27].

Digital messaging provides another potential source of dietary information for pregnant women and their families. In South Africa, MomConnect, the national maternal mobile messaging service of the Department of Health, registers women at their first antenatal visit and sends free stage-based messages on pregnancy, including nutrition. By 2018, it had reached the majority of women attending first antenatal care and was operating in almost all public health facilities [28]. User assessments report that women find the messages acceptable and trustworthy and act on some of them, and that they routinely relay the content to family members [29,30], whereas program evaluations document that reach and message exposure are considerably less even than facility coverage suggests [31,32]. The wider evidence on maternal mobile health (mHealth) in low-income and middle-income settings likewise finds effects concentrated on service contact and knowledge rather than on intake [33]. What is not established is how such messages fare once they enter households in which dietary decisions are already governed by elders.

This study explores how cultural and intergenerational power relations shape maternal dietary practices in rural South Africa. We examine: *1*) how elder authority influences pregnant women's food choices; *2*) how they navigate competing advice from healthcare providers, family elders and community leaders; *3*) what strategies they use to exercise agency within household hierarchies; *4*) how broader social and system-level constraints prevent women from putting nutritional knowledge into everyday practice; and *5*) how digital connectivity creates new spaces for knowledge exchange and influences what food ends up on the plate.

Two complementary frameworks guided this study. The Social Ecological Model (SEM) conceptualizes health behavior as shaped by interacting influences at the individual, interpersonal, community, institutional, environmental, and policy levels [34]. We used SEM to examine the different levels that influence maternal dietary practices, from women's knowledge and family relationships to community and health-system factors. Bourdieu's theory of practice was used to examine how household authority and power shape which sources of dietary knowledge are considered legitimate and followed [35]. We drew on 4 concepts: habitus, capital, field, and symbolic power. Together, these frameworks allowed us to examine both where influences on maternal diet operate and why some sources of advice carry greater authority than others within the household. Bourdieu's concepts have been applied to food and eating in a range of settings [[36], [37], [38]] and to women's health research in Africa, but rarely to maternal dietary decision making in rural sub-Saharan Africa.

## Methods

### Study design and setting

We conducted a qualitative study using focus group discussions (FGDs) and semistructured in-depth interviews (IDIs). The SEM [34] and Bourdieu’s Theory of Practice [35] were applied at the analytic stage and are described under Data analysis. The study took place in rural parts of Capricorn district, Limpopo Province, South Africa, which includes communities with marked socioeconomic variation. Capricorn was selected for 3 reasons. It records among the highest levels of household food insecurity and rural deprivation in the province; it falls within the catchment of the University of Limpopo, whose investigators on this study have established working relationships with the district health office, clinic managers, and traditional leadership through prior maternal health research; and it is the implementation site of the wider maternal nutrition program within which this study sits. These relationships enabled clinic-based recruitment and community entry through recognized gatekeepers. Eight public primary healthcare clinics were purposively selected, in consultation with district health officials, to represent geographic spread, service environments, and community characteristics.

### Participant selection and recruitment

Purposive sampling captured diverse caregiving and decision-making roles. Eight participant groups were sampled: pregnant women, grandmothers, fathers, community leaders, community health workers, mother mentors, professional nurses, and midwives. The eligibility criteria, the recruitment route, and the data collection method used for each group were specified in advance and are set out in Table 1. The predominant focus on pregnant women was preplanned, reflecting the study’s objectives. Two points warrant emphasis here. Grandmothers were included on confirmed advisory status rather than on age or kinship alone: on a single screening question put by the clinic-linked community health worker, each confirmed that she advised a currently or recently pregnant woman on what she should and should not eat. Mother mentors are lay peer counselors attached to primary care clinics in South Africa who provide one-to-one counseling, support, and referral to pregnant women and new mothers; they are distinct from community health workers, whose work is household based.

**TABLE 1:** Participant groups, eligibility criteria, recruitment route, data collection method, and composition

Participant group	Eligibility criteria	Recruitment route	Method	Sessions	Participants	Men	Women
Pregnant women	Aged 18 y or older; currently, pregnant; resident in the catchment of a participating clinic and attending antenatal care there	Approached at participating clinics by research assistants on antenatal clinic days	FGD	11	56	0	56
Grandmothers	Mother or mother-in-law of a woman currently pregnant or delivered within the preceding 12 mo, who confirmed on a single screening question that she advised that woman on what she should and should not eat	Clinic-linked community health worker networks and community leadership structures	FGD	2	21	0	21
Fathers	Male partner or father with a child with a woman currently pregnant or delivered within the preceding 12 mo, resident in the catchment of a participating clinic	Clinic-linked community health worker networks and community leadership structures	FGD	1	5	5	0
Community leaders	Man or woman recognized by community leadership structures as a community leader, resident in the community for ≥10 y	Community leadership structures	FGD	3	21	15	6
Community health workers	Employed as a community health worker linked to a participating clinic, with household-based outreach duties	Clinic-linked community health worker networks	FGD	5	34	6	28
Mother mentors	Lay peer counselor attached to a participating clinic providing one-to-one counseling, support and referral to pregnant women and new mothers	Approached at participating clinics by research assistants	IDI	5	5	0	5
Professional nurses	Professional nurse with ≥6 mo of maternal health experience at a participating clinic	Approached at participating clinics by research assistants	IDI	5	5	0	5
Midwives	Midwife with ≥6 mo of maternal health experience at a participating clinic	Approached at participating clinics by research assistants	IDI	3	3	0	3
Total				35	150	26	124

All participants received a participant information sheet in Sepedi and were contacted subsequently to arrange a session. Sampling sought variation in age, parity, and position within the household, by which we mean whether a participant purchases the food, prepares it, or has it prepared for her, because it is these positions rather than employment status that determine influence over the diet.

A total of 586 individuals were approached and invited to participate, of whom 150 agreed and took part, whereas 436 declined or were unable to participate. Self-reported reasons for declining were recorded at the point of refusal for every individual approached and are summarized in the recruitment flowchart (Figure 1).

**FIGURE 1:**
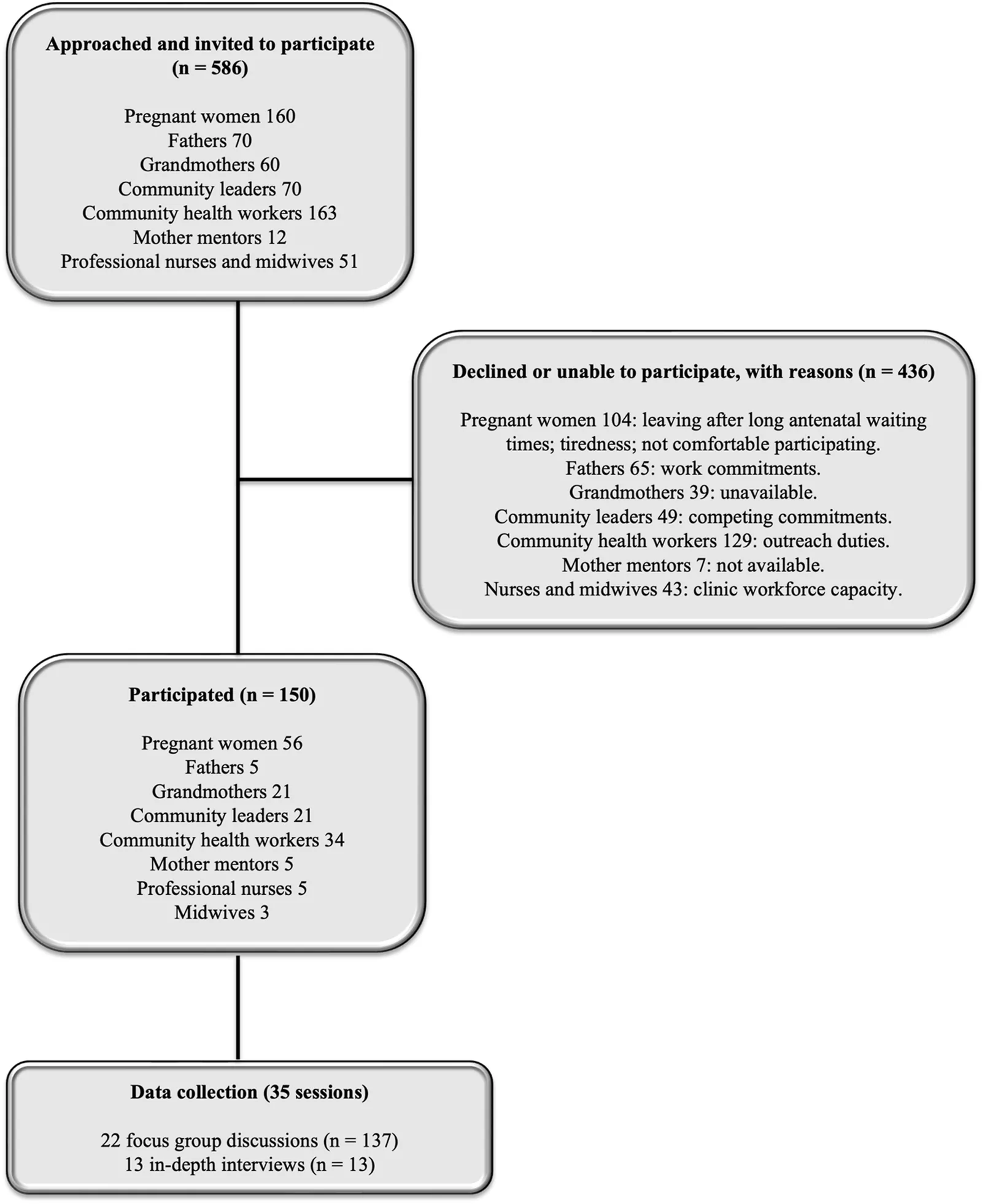
Study participant recruitment flowchart.

### Data collection

Between March and October 2025, 22 FGDs (5–8 participants, 60–90 min) and 13 IDIs (45–75 min) were conducted, engaging 150 participants. The choice of method followed the object of interest. FGDs were used with community members, where the object of interest was the collectively held norm and the manner in which dissent about food is voiced or withheld before others; each group was composed of participants sharing a single role, because candid discussion of elder authority is unlikely in the presence of elders. IDIs were used with mother mentors, nurses, and midwives, where the object of interest was professional experience of countering household advice, where confidentiality was material, and who were few in number at any one clinic.

Sessions were conducted in Sepedi by the 3 trained research assistants, with a second research assistant present at every session as note taker. No other person was present. Eight semistructured guides were used, one for each participant group: pregnant women, grandmothers and grandfathers, fathers, community leaders, community health workers, mother mentors, professional nurse, and midwives. The guides were developed collaboratively by the research team and are provided in Supplemental File 1. Each opened with a short demographic section and then covered 6 content domains. The domains were derived from the study objectives by taking each objective in turn and specifying the everyday matters a participant could speak to that would bear on it. They were: *i*) understanding of maternal nutrition and current dietary practice, from objective 1; *ii*) sources of dietary advice and the roles of household members in food decisions, from objectives 1 and 2; *iii*) cultural beliefs, traditions and food taboos, from objectives 1 and 2; *iv*) antenatal counseling, nutrition programs and supplements, from objectives 2 and 3; *v*) access to nutritious food and to services, including what participants thought could be done to improve it, from objective 4; and *vi*) use of mobile phones and digital information, from objective 5. Wording was adapted to each group, so that pregnant women were asked what they themselves ate and were advised, whereas grandparents, fathers, community leaders, community health workers, and providers were asked about the part they played in what a pregnant woman ate. We did not assign questions to levels of the SEM [34]. Influences frequently sit at more than one level, and the same matter may be individual in one account and institutional in another: whether a supplement is taken is a matter for the woman and her household, whereas whether it is recommended and supplied is a matter for the clinic. The SEM was therefore used at the analytic stage to locate influences rather than at the design stage to allocate questions.

The guides were piloted in a nonparticipating clinic and refined for cultural and linguistic clarity. All discussions were audio-recorded after written consent, with field notes capturing context and nonverbal cues.

### Translation and data management

Recordings were transcribed verbatim in Sepedi and translated into English by bilingual assistants. To ensure conceptual accuracy, 20% of transcripts were back-translated by independent reviewers, with discrepancies resolved through team consensus. All transcripts were anonymized and stored on encrypted, password-protected drives.

### Data analysis

Analysis followed Braun and Clarke’s 6-phase reflexive thematic framework [39]. The 6 phases comprised familiarization through repeated reading; generation of initial codes across the full dataset; collation of codes into candidate themes; review of themes against the coded extracts and the dataset as a whole; definition and naming of themes; and production of the report. Coding was primarily inductive and no codebook was prepared a priori. NVivo 12 supported data organization. Coding was applied to the full transcripts rather than domain by domain, so that material elicited under one content domain could be coded to any theme.

Two researchers independently open-coded 8 transcripts spanning pregnant women, grandparents, community health workers, and mother mentors, compared codes line by line, and consolidated the agreed codes into a working codebook. The remaining transcripts were coded against that codebook, with new codes added inductively. Candidate themes were generated by grouping codes that shared an underlying meaning rather than a shared topic. Accounts were compared across participant groups so that convergence and divergence could be established rather than assumed. Every quotation reported below was verified against the source transcript and is identified by participant group and session. Quotations were selected for analytic relevance rather than to illustrate every code, and only those extracts that carry the interpretation are retained in the text. Regular peer debriefing supported dependability, and an audit trail documented coding iterations and analytic decisions.

Two frameworks guided interpretation. The SEM [34] locates where influence on the diet is exerted, across individual, interpersonal, community, institutional, and policy levels. Bourdieu's Theory of Practice [35] explains why some sources of dietary knowledge are treated as authoritative within the household and others are not, through habitus, capital, field, and symbolic power. Both were applied after phase 5, once the themes had been developed and defined, and were used to interpret the themes rather than to generate the initial codes. Their application had 3 steps. First, each theme was located at the level or levels of the SEM at which the influence it described was exerted; the criterion was the content of the account rather than the participant group, so that a provider's account of household decision making was located at the interpersonal level. Second, each theme was re-read against Bourdieu's concepts, and the concept retained for each theme was the one that accounted for the largest number of extracts within that theme. Elder authority was read as embodied cultural capital where participants grounded it in reproductive experience rather than in instruction or credential, as in the statement “I carried ten children, I know better.” Public deference was read as habitus where compliance was described as a matter of course rather than as a decision. Divergence between what women said at the clinic and what they did at home was read as movement between fields, the criterion being that the same woman was reported to hold one position in one setting and another elsewhere. Material deprivation was read as a constraint of economic capital that limits the translation of knowledge into practice. Restriction framed as care was read as symbolic power where the person imposing it was also the person providing care, so that refusal carried a relational rather than a nutritional cost. Third, extracts that did not fit the retained concept were examined rather than set aside; the midwife’s account of a generational loosening reported under subtheme 4.2 is retained for this reason, because was not corroborated in the accounts of grandmothers or pregnant women. The resulting mapping of themes to SEM levels and to Bourdieusian concepts is shown in Figure 2.

**FIGURE 2:**
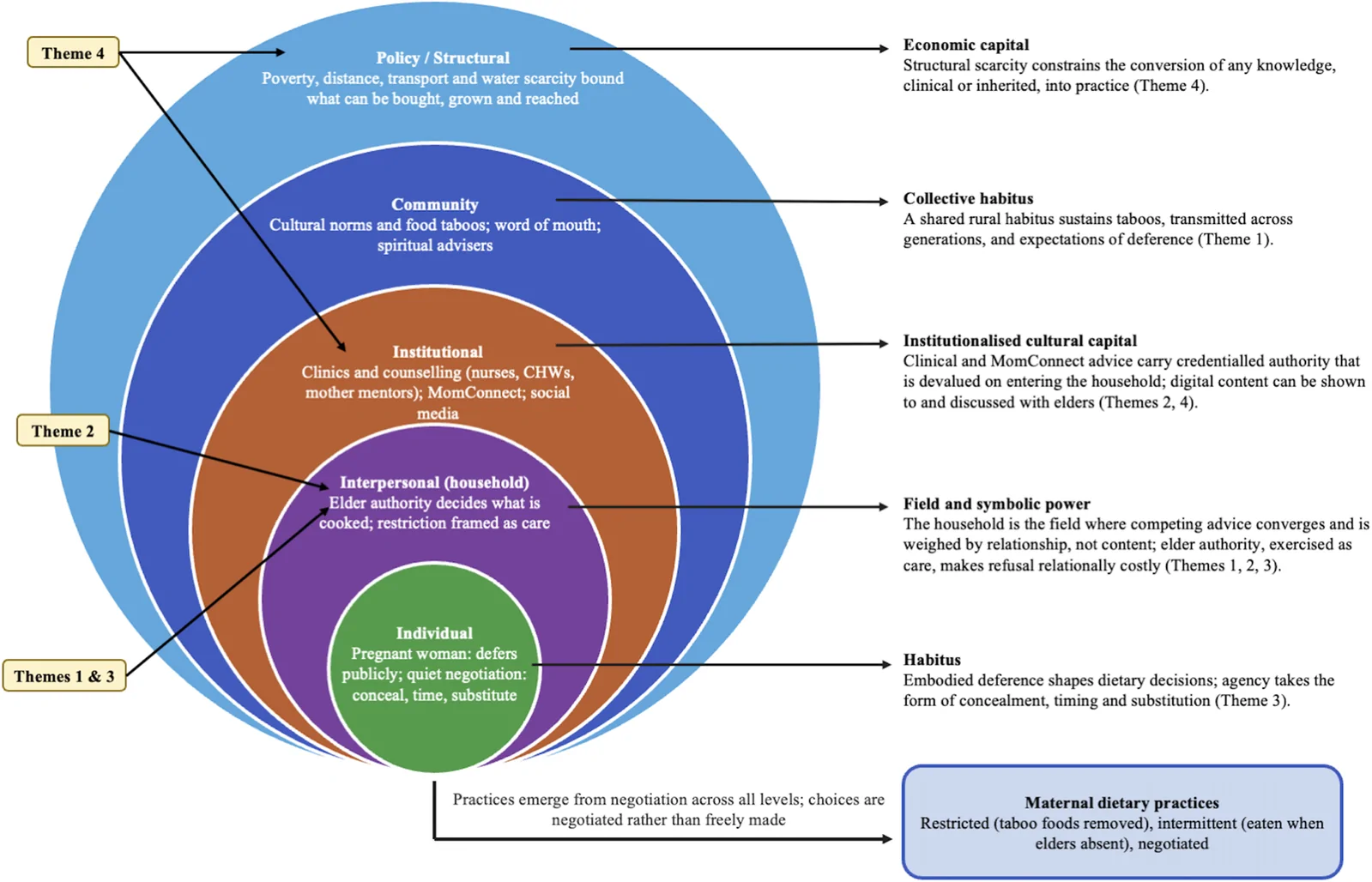
Integrated conceptual model of maternal dietary practices, combining Social Ecological Model levels (rings) with Bourdieu's concepts (right) and the location of the 4 study themes (left). Themes: *1*) intergenerational authority and moral power; *2*) negotiating between traditional and clinical advice; *3*) maternal agency under intergenerational constraint; *4*) contextual barriers and changing dynamics. CHW, community health worker.

### Ethical considerations

The study was conducted in accordance with the ethical principles of the Declaration of Helsinki. Ethical approval was obtained from the Turfloop Research Ethics Committee, University of Limpopo (Ref. TREC/XXX/2025:PG). Permissions were secured from the Limpopo Provincial Department of Health, the Capricorn District Health Office, and all clinic managers. Participation was voluntary, with written informed consent and assurance of anonymity and withdrawal rights.

### Data sharing and reporting

De-identified excerpts are available from the corresponding author upon reasonable request, in line with institutional governance procedures. Reporting followed the Consolidated Criteria for Reporting Qualitative Research, and the completed checklist is provided with this submission.

### Researcher reflexivity

Data were collected by 3 bilingual trained data collectors fluent in Sepedi and English, 2 women and 1 man, none of whom were affiliated with the participating clinics. All researchers involved in the research project had prior training and experience in qualitative interviewing and community-based research. Participants were informed of the researchers’ roles, the purpose of the study, and the voluntary nature of participation. Researcher gender, language, and professional background were recognized as potential influences on interactional dynamics, particularly in discussions related to maternal nutrition. Reflexive journals and weekly debriefings with senior investigators were used to support ongoing reflexivity and analytic consistency. We report the gender of research team members and of participants rather than their sex, because the analytic interest lies in gendered authority and in the bearing of a facilitator's gender on what participants felt able to say.

## Results

Thematic analysis identified 4 themes and 8 subthemes that guided the analysis (Table 2). Together, these themes describe how cultural authority, competing sources of knowledge, and material constraints shape the foods that pregnant women report eating and avoiding.

**TABLE 2:** Themes and subthemes

Theme	Subthemes
1. Intergenerational authority and moral power	1.1 Elder authority in maternal diet
	1.2 Intergenerational transmission of taboos
2. Negotiating between traditional and clinical advice	2.1 Competing home and clinic guidance
	2.2 The fate of specific clinical recommendations
3. Maternal agency under intergenerational constraint	3.1 Normative obedience and deference
	3.2 Quiet negotiation and microresistance
4. Contextual barriers and changing dynamics	4.1 Material constraints on dietary practice
	4.2 Digital platforms create supplementary authority

Accounts converged and diverged in a patterned way, and this patterning is reported throughout. Pregnant women, grandmothers, fathers, community leaders, community health workers, mother mentors, professional nurse, and midwives all described elder authority over the diet as real and as consequential; they differed on how they evaluated it. Grandmothers and community leaders described it as legitimate and protective.

Healthcare workers and mother mentors described it as an obstacle to antenatal advice. Pregnant women described it as both, accepting its legitimacy while recounting the strategies by which they worked around it.

### Theme 1: Intergenerational authority and moral power

Family elders, especially grandmothers and mothers-in-law, shaped pregnant women’s diets through advice grounded in experience and viewed as protective. Two subthemes capture this influence: (1.1) elder authority in maternal diet, and (1.2) intergenerational transmission of taboos.

### Subtheme 1.1: Elder authority in maternal diet

This subtheme concerns the basis and legitimacy of elder authority, that is, who is entitled to determine what a pregnant woman eats and why that entitlement is accepted. Elder women, principally mothers-in-law and grandmothers, held the decisive say over what was cooked and served. Their authority rested on reproductive experience rather than on formal education, and it was exercised through command of the kitchen rather than through instruction. In every account in which elder authority was described as bearing on food, its effect was to remove items from the pregnancy diet rather than to add them, and no participant described an elder requiring that a nutrient-dense food be eaten. The foods withheld under elder authority were the nutrient-dense animal-source and green leafy foods that antenatal counseling promotes, chiefly eggs, spinach, and pumpkin, and the effect described was reduced intake of these foods rather than substitution of a nutritional equivalent.

"We tell them not to eat eggs because we believe it makes the baby slow to speak. It is not that eggs are bad, but that we have seen children take longer to talk when their mothers eat too many eggs during pregnancy. We do not want to take chances. Even when the clinic says they are good, most women here will still avoid them. These are things that have been passed from our parents, and we continue them because we want our daughters and grandchildren to be safe" (Grandmother, FGD 1).

"When a woman is pregnant, she must listen to the old people. They have experience. I live with my mother-in-law, and she cooks for me. She chooses what I eat and what I don’t eat. Even when I hear something different at the clinic, I keep quiet because she says, 'I carried ten children, I know better.' I respect her because she takes care of me" (Pregnant Woman, FGD 9).

This authority extended in some households to the clinical encounter itself. Community health workers described elders positioning themselves between the pregnant woman and the health system, which is a more concrete mechanism than the diffuse cultural influence usually invoked.

"There was an instance where I was trying to consult with a pregnant woman, but the grandmother of the household insisted on being present during the conversation. She was resistant to the idea of anyone speaking with the pregnant woman without her being there" (Community Health Worker, FGD 3).

"Those who are willing to give us an ear or open up might be about 5 per cent, or those who are not living with elders at home, especially the youth" (Community Health Worker, FGD 2).

### Subtheme 1.2: Intergenerational transmission of taboos

Whereas subtheme 1.1 concerns why elders are obeyed, this subtheme concerns what is transmitted and by what means. Elders acted as custodians of a classificatory system in which foods carry properties, notably heat, and in which particular harms are attributed to particular foods. The rules named by participants were consistent across groups: eggs were linked to delayed speech, to an oversized fetus and to cord entanglement; spinach to discolored skin; pumpkin and carrots to heat and to infant rashes; and firm foods to obstructed labor. Transmission occurred through instruction during cooking, through cautionary narratives about named children, and through direct sanction of dissenters, including the health workers who advised otherwise. The dietary consequence is the removal of eggs, green leafy vegetables, and orange-fleshed vegetables from the pregnancy diet, which in these households means the removal of the principal accessible sources of protein, iron, folate and provitamin A.

"They avoid pumpkin and carrots because they are hot foods. We believe that when a woman eats too much of these, it brings out heat in the womb, and that heat gives the baby skin problems like rashes. We learned this from our mothers and we teach our daughters." (Grandmother, FGD 2).

"One time I told a girl to eat more eggs, but her grandmother got angry with me. She said, 'Who are you to change our ways?' The girl was scared to go against her grandmother. She told me she would rather stop the eggs than cause a fight at home. So, she stopped again, even though her iron was low" (Mother Mentor, IDI 4).

### Theme 2: Negotiating between traditional and clinical advice

This theme describes the contradictory field of advice within which pregnant women act: where dietary guidance comes from, how households weigh it, and what happens to clinical recommendations once they enter that field. Two subthemes capture this: (2.1) competing home and clinical guidance, and (2.2) the fate of specific clinical recommendations.

### Subtheme 2.1: Competing home and clinic guidance

Dietary advice reached pregnant women from many directions at once: the clinic, family elders, husbands, community word of mouth, and, in the accounts of mother mentors, prophets, and traditional healers who prescribed practices such as avoiding salt. Three features of this field recur across the quotes. First, sequence: community and family advice arrives before clinical advice, so the clinic speaks into a field already occupied. Second, arbitration: when sources conflict, the decision is made inside the household, not at the clinic, and it is often made by someone other than the pregnant woman herself. Third, and most consequentially, advice is weighed by the relationship and future obligation of the adviser rather than by its content. The grandmother's own explanation is explicit on this point: her word prevails not because it is judged more accurate but because she will be present at the labor and after the birth, when the nurse will not. The father's account shows the same logic from another position in the household: he does not judge the clinic wrong, he judges the cost of contradicting his mother too high.

"They have friends and families who talk to them before we even come. By the time we reach, they are clouded by the myths related to maternal nutrition" (Community Health Worker, FGD 5).

"Even though the nurse said pumpkin is healthy, the daughters still listen to us, because we will be there with them during labour and after the baby is born." (Grandmother, FGD 2)

"My wife tells me what the clinic says, but my mother is firm. She says, 'In our house, a pregnant woman doesn't eat eggs or bananas.' I tell my wife to listen to her because I don't want arguments. The nurses may be right, but at home my mother's word counts. I think both sides want the same thing, a healthy baby, but in different ways." (Father, FGD 1).

### Subtheme 2.2: The fate of specific clinical recommendations

This subtheme follows 2 named clinical recommendations into the household and asks what became of each; they failed in different ways. Antenatal supplements were widely believed to enlarge the fetus, and providers reported that women accept the tablets at the clinic and then do not take them, so that the routinely recorded indicator of adherence, collection, diverges from consumption. The promotion of eggs and meat failed for a different reason: the recommendation itself was not disputed, but a woman who repeated it at home was heard as claiming to know better than her elders, and the price of that claim was conflict. In neither case was the mechanism ignorance; women who could state the advice accurately declined to act on it. A third feature concerns the messenger rather than the message: community health workers reported being dismissed on account of their rank, with households insisting on hearing the same advice from a nurse, so that the standing of the adviser governed the fate of the advice. Providers described responding with awareness sessions on antenatal care days and one-to-one counseling, but their own accounts locate the limit of this strategy: as one mother mentor put it, "we explain, but there are myths that are rooted in their heads."

"Problem with our people is that when you advise them to take the supplements given at the clinic, they say the pills make the tummy grow bigger. They still have mindset of saying if we take pills from the clinic, we will have big tummies. So, they collect them, but they don't drink them" (Professional Nurse, IDI 2).

"When I came back from the clinic, I told my mother what the nurse said about eating eggs and meat. She looked at me and said, 'So now you know more than us?' I didn't say anything after that… If I want peace in this house, I must listen." (Pregnant woman, FGD 3)

"Sometimes they undermine us because we are just community health care workers; they need the nurses to talk to them about maternal nutrition" (Community Health Worker, FGD 4).

### Theme 3: Maternal agency under intergenerational constraint

This theme describes the agency available to pregnant women within hierarchical households: why open contestation is foreclosed, and the discreet strategies through which clinic advice is partially followed nonetheless. Two subthemes emerged: (3.1) normative obedience and deference, and (3.2) quiet negotiation and microresistance.

### Subtheme 3.1: Normative obedience and deference

Open disagreement with an elder over food was described as unavailable to pregnant women, for 2 reasons that the quotes distinguish. The first is normative: deference to elders is a community rule, stated flatly by a community leader as "the young do not question the elders because they respect them." The second is relational and reciprocal: the elder who restricts the diet is also the person who buys, cooks, and serves the food and who cares for the pregnant woman daily, so refusal reads as ingratitude toward a caregiver rather than as a nutritional choice. The consequence for counseling is visible in a scene community health workers described as routine: the elder answers on the pregnant woman's behalf during group talks, whereas the younger woman nods, then privately tells the health worker that she knows what to do but cannot do it at home. Silence in the clinic is therefore not agreement, and not ignorance; it is deference performed in public, and it means that assent recorded during counseling cannot be read as adherence.

"I live with my mother-in-law, and she takes care of me. She makes sure I eat and rest. When she says something is not good for me, I listen because she is the one looking after me. It is hard to say no to the person who cooks for you" (Pregnant Woman, FGD 4).

"Sometimes when we give group talks, the older woman answers for the pregnant one. The younger woman just nods. After the talk, she tells me quietly that she knows what to do but cannot do it at home because her mother or grandmother will shout. That is what we deal with daily" (Community Health Worker, FGD 2).

### Subtheme 3.2: Quiet negotiation and microresistance

Because open refusal was foreclosed, agency took discreet forms, and the quotes specify 3 tactics with distinct dietary consequences. The first is concealment: supplements are hidden and taken at night, preserving micronutrient intake by removing it from household view. The second is timing: clinic-recommended foods are eaten during the elder's absence, so that adherence is episodic and governed by household composition rather than by conviction. The third is substitution: a prohibited food is replaced by a permitted one of comparable values, beans or chicken standing in for eggs. Substitution was not invented by the women alone; it was actively coached by mother mentors, and it is the only tactic in our data that recovers protein intake without requiring secrecy.

"At home they say no tablets, but at the clinic they give them to me. I keep them in my bag and take them at night. I do not tell anyone. It is better that way. If I say I am taking them, they will shout at me. So, I keep quiet and take them" (Pregnant Woman, FGD 4).

"Sometimes I eat what the nurse said when my mother-in-law is away. I do not want to argue with her. She believes it is dangerous. I just try to balance both. When she is here, I follow her way. When she is gone, I try to eat what I was told at the clinic" (Pregnant Woman, FGD 11).

"We tell them that they can still respect their elders but also take care of themselves. We say, 'If granny says no to eggs, then eat beans or chicken.' We help them find something in between so that the home is peaceful and the baby is still healthy. That is how we survive these situations" (Mother Mentor, IDI 5).

### Theme 4: Contextual barriers and changing dynamics

This theme situates maternal diet within material deprivation and emerging digital influences that reshape, but do not replace, elder authority. Two subthemes emerged: (4.1) material constraints on dietary practice, and (4.2) digital platforms create supplementary authority.

### Subtheme 4.1: Material constraints on dietary practice

Three material constraints were described, each bearing on the diet in a different way. The first is affordability. Participants in every group reported that the foods promoted in antenatal counseling, namely meat, milk, eggs, and fruit, are precisely the foods forgone when cash is short; as one pregnant woman put it simply, "when you don't have money, you won't be able to buy anything you need." The second is water: tanked water rationed to domestic use forecloses household food gardening, removing the only route by which these households might obtain vegetables without cash. The third is distance and the cost of transport, which determine whether counseling is received at all, because, as a community leader and a midwife both observed, a woman who cannot reach the clinic misses both the supplement and the message. The quotes also reveal a consequence that runs deeper than unaffordability: material constraint reshapes the counseling encounter itself. Community health workers reported abandoning the topic of food altogether in the poorest households, and the midwife's account of being asked "Sister, will you buy it for me?" shows dietary advice collapsing into an unanswerable question about money. The constraint therefore operates twice, once on what households can buy and once on what health workers feel able to say. Finally, these constraints do not compete with elder authority so much as compound it: where nutrient-dense foods are unaffordable in any event, a taboo prohibiting them costs the household nothing to observe and is never put to the test.

"Sometimes we stand in front of a group and talk about eating fruits, milk and meat, but you can see from their faces that they cannot afford it. One woman said to me, 'Sister, will you buy it for me?' and the whole room laughed. That moment tells you that knowledge is not the problem money is. We leave feeling helpless because our advice alone cannot fill an empty plate" (Midwife, IDI 2).

"We try, but unfortunately, because of poverty in some houses, we end up not talking about food" (Community Health Worker, FGD 3).

"We struggle with water... it's delivered by tankers and only for domestic use. You can't grow anything with that" (Pregnant Woman, FGD 4).

### Subtheme 4.2: Digital platforms create supplementary authority

MomConnect is the national maternal mobile messaging service of the South African Department of Health. Women are registered at their first antenatal visit and receive free stage-based text messages covering pregnancy, including nutrition, with access to a query helpdesk [29]. Participants distinguished between MomConnect and other digital content. MomConnect messages were described as aligned with clinic advice and as carrying the clinic's authority into the home, and mother mentors reported that they prompted reconsideration of specific prohibitions, most often the prohibition on eggs. Content encountered on Facebook, YouTube, and WhatsApp was described as a source of ideas about food rather than of instruction. What distinguishes digital information from a nurse's advice in these accounts is its mode of entry into the household. A message or picture can be shown to an elder and discussed; community health workers described the phone becoming "a new meeting point for old and young" when they circulated pictures of healthy food and gardens. The grandmother's own account is telling: she looks at the pictures her granddaughters show her and laughs, engaging with the content as a curiosity rather than rebuking it as a challenge, in contrast to the direct sanction that health workers who contradicted the taboos received (subtheme 1.2). Digital content is tolerated precisely because it does not present itself as a rival authority, and for the same reason it displaces little: we found no account of digital content promoting a harmful dietary practice, and equally no account of a woman resolving a disagreement with an elder by invoking a message. One midwife perceived a broader generational loosening, reporting that the myths "are weaker" and that younger women "still respect their mothers, but they decide for themselves"; this was a single provider's assessment and was not corroborated in the accounts of grandmothers or pregnant women. Access, finally, was constrained by cost twice over: pregnant women described being unable to afford the data to view food content, and unable to afford the foods it showed. Our data speak to affordability among participants who already own phones and do not permit any claim about the distribution of connectivity or handset ownership in the district.

"Most of the pregnant women we visit already know about diet from MomConnect. When you ask them what to eat, they say, 'We get the messages on our phones.' They like it because it reminds them to eat fruits and to take the supplements. Even when the grandmother says, 'Don't eat eggs,' the messages say, 'Eat eggs for protein.' So they think about it and sometimes they follow what is in the message" (Mother Mentor, IDI 5).

"Things are different now. These girls have phones, and they get messages from the clinic. Sometimes they show me pictures of food on WhatsApp and say, 'this is what they say we must eat.' I look at it and laugh because in our time we did not have such things." (Grandmother, FGD 2).

"I check Facebook or YouTube for food ideas, but data is costly, and I can't always afford what they show" (Pregnant Woman, FGD 7).

## Discussion

The main findings of this study are that maternal dietary practices in rural South Africa are shaped by grandmother authority exercised as care, by strategic negotiation on the part of pregnant women, by material deprivation that sets the limits within which both operate, and by digital messaging that enters this arrangement as an additional but subordinate source of dietary authority. Grandmothers and mothers-in-law held decisive control over food choices, and framed their advice as care and protection. Pregnant women knew the clinical advice but practiced public deference and private adaptation to maintain family harmony. Poverty, inadequate infrastructure, and limited water access constrained what either inherited or clinical knowledge could be converted into, so that dietary advice was frequently received as aspirational. Digital messages aligned with clinical advice circulated widely and were discussed across generations, but did not displace elder authority over what was cooked.

### Interpretation through the Social Ecological Model and Bourdieu’s theory of practice

Maternal dietary practice in this sample emerged from intersecting layers of influence rather than from individual preference, knowledge, or ability alone. Our themes map directly onto the SEM levels set out in the Introduction, and Figure 2 shows this mapping alongside the Bourdieusian concept operating at each level. Themes 1 and 3 operate at the interpersonal level, where elder authority is exercised and deference performed. Theme 2 sits at the boundary between the interpersonal and institutional levels, where household and clinic advices collide. Theme 4 spans the institutional and structural levels, where counseling, digital messaging, income, water, and transport shape what any advice can achieve. In this structure, choices are negotiated rather than freely made. Individual-level knowledge alone appeared insufficient to explain reported dietary practice, with participants repeatedly describing interpersonal and structural constraints on whether clinical advice could be enacted.

Bourdieu's theory explains why some knowledge commands the plate and other knowledge does not. The clinic and the household operate as distinct fields, each with its own dominant capital. In the clinic, authority rests on institutionalized cultural capital, conferred by professional credentials. In the household, the operative capital is embodied reproductive experience. Participants stated this in exactly those terms: "I carried ten children, I know better." Capital does not transfer freely between fields.

Clinical advice therefore lost its value at the household threshold, where repeating the nurse's guidance was heard as claiming to know better than one's elders. Elder authority also worked as symbolic power. Restriction was framed as care by the person who buys, cooks, and will attend the birth. Compliance therefore became gratitude, and refusal became disrespect. This is why no nutritional argument offset the relational cost of dissent. Habitus captures the embodied nature of this deference, visible in women who assented publicly during counseling while privately holding contrary knowledge. The tactics in subtheme 3.2, concealment, timing, and substitution, are strategic practice within the field rather than change of it. Women navigated skillfully without altering its rules, which is why none of these tactics changed what was habitually cooked. Economic capital sets the final limit. Without money, water, and transport, neither clinical nor inherited knowledge converts into practice.

Bourdieu’s concepts have been used to study food and eating for some time, so the contribution here is one of setting rather than of theory. A narrative review identified 38 studies applying concepts from distinction to food choice and provisioning, taste, social class, and food symbolism [36], a systematic review has examined cultural capital as a determinant of food choice [38], and Morais Sato et al. [37] used habitus, capital, and field to account for the eating practices of low-income Brazilian mothers. In African settings, the concepts have been used chiefly to examine women’s access to health services rather than their diets [40]. What is less common is their application to maternal dietary decision making in rural sub-Saharan Africa, where the operative capital is reproductive experience rather than class position and where the competing authority is a health service rather than a market. In our data, elder women’s experiential knowledge functioned as embodied cultural capital that outweighed the institutionalized capital of health providers within domestic space. Pregnant women’s habitus, formed through lifelong socialization into deference, predisposes them to comply even when they hold contrary clinical knowledge. This helps explain the intergenerational persistence of restrictive practices: they are reproduced through authority, obedience, and material deprivation and constrained living conditions, not through lack of information.

Digital knowledge is not uniform in its legitimacy. MomConnect borrows the clinic's legitimacy, because women are enrolled at the antenatal visit and recognize the health service as the sender. Its messages carry the clinic's authority into the home. Social media content carries no such warrant and was treated as suggestion rather than instruction. Both forms share one feature: their legitimacy is conferred outside the household, and this limits them within it. Digital content nonetheless enters the domestic field differently from a nurse's instruction.

A message or picture can be shown to an elder and discussed. It arrives as an object of shared interest, not a rival claim to her authority. This is why grandmothers met it with curiosity rather than the sanction directed at dissenting health workers, and why it supported dialog without displacing elder capital. Among participants who used these platforms, the cost of data limited acting on what they saw. Together, these findings indicate maternal diet as a socially regulated practice shaped by legitimacy, constraint and survival. What is eaten in pregnancy depends less on what women know than on whose knowledge counts, and on what the household can afford.

Our findings add to existing evidence by showing that maternal dietary practices in sub-Saharan Africa by showing that they emerge through active negotiation within intergenerational power structures rather than passive adherence to cultural tradition [25,[41], [42], [43], [44], [45]]. Framing elder dietary guidance as care and protection explains why pregnant women accept restrictions that contradict clinical advice. Moral framing transforms dietary restriction into an expression of familial love, so that resistance reads as disrespect, which complicates health promotion. Our findings identify a further mechanism: elders did not only advise but in some households gatekept the clinical encounter itself, insisting on being present when health workers spoke with pregnant women.

Our findings align with studies from Nepal, Colombia, Sierra Leone, and Kenya [43,[45], [46], [47]] showing grandmother influence, but extend this by showing authority legitimized through care rather than coercion [16]. The fact that pregnant women describe elder authority as protective rather than oppressive challenges deficit-based models that treat traditional practices simply as barriers. The dietary taboos identified mirror patterns across sub-Saharan Africa [23]. Their transmission through everyday instruction, cautionary narrative, and sanction of dissenters explains why these beliefs persist despite widespread clinical nutrition education, and why knowledge-transfer interventions alone may be insufficient [48].

Our findings on digital platforms are consistent with, and help to explain, the evaluation evidence on MomConnect. The program has achieved unusual scale, reaching the majority of women attending first antenatal care across almost all public facilities [28], and users find the messages acceptable and trustworthy, act on some of them, and relay their content to family members [29]. Evaluations nonetheless document that individual reach and message exposure are less even than facility coverage implies [31]. Our findings suggest a mechanism that routine program monitoring may not capture: the message arrives in a household where the person who received it is not the person who cooks. Our participants, like those of Skinner et al. [29], shared message content with relatives, but sharing is not prevailing, and no participant described a message settling a disagreement with an elder in the woman’s favor. This qualifies the assumption in the mHealth literature that better information flow to the individual woman will change intake, and it aligns with evidence that maternal mHealth shifts service contact and knowledge more reliably than diet [33]. It also yields a design implication: messages addressed to, or designed to be shown to, the household member who controls the kitchen would engage the level at which decisions are actually taken and would bring digital messaging into line with the grandmother-inclusive programming evaluated elsewhere in the region [[49], [50], [51]]. Two features of our data support feasibility. Digital messaging was consistently aligned with clinical rather than restrictive advice, and it was the only counter-authority grandmothers willingly engaged with, being something that can be looked at and discussed rather than an instruction issued by a younger professional.

The concept of quiet negotiation aligns with feminist scholarship on women’s agency under structural constraint [52] and parallels research from South Asian contexts [53]. The finding that advice framed as care outweighs scientifically grounded recommendations resonates with research on vaccine hesitancy and other health behaviors [54]. Health promotion in these settings is relational before it is informational. The rank of the messenger mattered too: households dismissed community health workers and insisted on nurses, which bears directly on the task shifting of maternal health interventions that the WHO recommends as a means of extending access [55] and on the counseling components of its antenatal care guidance [56].

Cultural authority and material deprivation were not competing explanations in these accounts but compounding ones. The role of food insecurity in constraining maternal diets is well documented [57,58] and aligns with global concerns regarding poverty and hunger, as reflected in the first 2 Sustainable Development Goals [59]. The distinctive contribution of our findings concerns cultural authority, which was described across all participant groups and appeared to provide the mechanism through which nutrient-dense foods were withheld. Material deprivation was described more narrowly, chiefly by healthcare providers and under subtheme 4.1, and it determined the scope within which that mechanism operated. The relation between the 2 is the more consequential observation. Where meat, eggs, and fruit are unaffordable, a prohibition on them costs nothing to observe and is difficult to falsify, because a well-nourished pregnancy is seldom available in the household as a counterexample. Poverty also reshaped counseling itself: health workers reported abandoning the topic of food in the poorest households. Cultural restriction and material scarcity are therefore mutually reinforcing rather than rival, and an intervention addressing only knowledge fails twice over, once because knowledge is not decisive and once because the recommended foods cannot be bought. This supports arguments that behavior change alone does not address the structural causes of health inequities.

### Strengths and limitations

The principal strength of this study is a multistakeholder design that allowed accounts of the same practices to be compared across the people who give, receive, and contest dietary advice. Several limitations qualify the findings. Social desirability may have led participants to emphasize harmonious relationships, and the deference described in subtheme 3.1 makes it likely that pregnant women understated their own dissent, particularly in groups. Much of the evidence on how women navigate competing advice comes from observers rather than the women themselves, which limits claims about their reasoning. Participation rates differed sharply between groups, and the low participation of fathers and of clinical staff limits what we can say about their perspectives; the recorded reasons for declining were predominantly practical rather than topic-related (Figure 1), but we cannot exclude that those who declined held different views about elder authority. Recruitment through clinics excluded women who do not attend antenatal care, who are likely to be the most constrained. Dietary data were wholly self-reported and not accompanied by dietary assessment, so we speak to reported practice, not intake, dietary diversity or nutritional status. We collected no data on connectivity or handset ownership and make no claim about their distribution. Findings are transferable to comparable rural, kin-dense settings in South Africa rather than generalizable beyond them.

Policy implications: participants were asked directly what they thought would improve access to an adequate diet in pregnancy, as part of content domain (v), and providers were asked what they already did when household and clinic advice diverged. The recommendations below draw on those responses as well as on our own analysis, and we indicate which is which. The implications follow the compounding relationship described above: household-level and structural measures are complements, not alternatives.

First, interventions should engage households rather than individual women, and particularly the grandmothers and other elders who decide what is cooked, because counseling directed only at pregnant women targets the weakest level of influence in our data. This follows from our analysis rather than from a recommendation made by participants. This aligns with grandmother-inclusive programming elsewhere in sub-Saharan Africa. In Senegal, engaging grandmothers as advisers rather than treating them as obstacles was associated with improved maternal and child nutrition practices [51], and in rural Sierra Leone a grandmother-inclusive approach was associated with improved infant and young child feeding practices [50]. The broader case for a family system perspective in nutrition programming is set out by Aubel et al. [49].

Second, counseling practice should assume that public assent is not adherence. This recommendation rests on practices participants already described rather than on proposals of our own. Providers in this study already described responding to competing advice with awareness sessions on antenatal care days and with one-to-one counseling where group sessions did not land, and mother mentors described coaching the substitution of permitted foods for prohibited ones. Supporting and formalizing these existing practices is more feasible than introducing new ones, and it is consistent with the recommendation that nutrition counseling in antenatal care be adapted to context and that components of antenatal care be task-shifted with appropriate support [56].

Third, digital messaging should be designed for the household rather than for the individual recipient, so that content can be shown to and discussed with elders. This is our inference from the pattern reported in subtheme 4.2 rather than a participant suggestion, and the grounds for thinking it feasible are set out above.

Fourth, none of these substitutes for action on poverty, food insecurity, water, and transport. Participants themselves were explicit that these determine whether any advice can be followed, and health workers described dietary advice collapsing into an unanswerable question about money. Health services alone cannot improve population health or reduce health inequities [60], and framing that stops at individual behavior leaves the social determinants of these practices untouched [61].

## Conclusion

Maternal dietary practices in rural South Africa emerge through negotiations within hierarchical households, constrained by material deprivation and increasingly mediated through digital channels. Grandmothers exercise authority grounded in experiential knowledge and moral obligation, whereas pregnant women navigate competing guidance through public respect and private adaptation. Material deprivation determines the scope within which that authority operates, so that cultural restriction and material scarcity compound rather than compete. Effective interventions require multi-generational and multisectoral approaches, combining engagement of household decision makers with social protection and infrastructure development that make an adequate diet attainable.

## Author contributions

The authors’ responsibilities were as follows – PA: conceptualized the study, led the analysis, and drafted the manuscript; LM, NM, FA-M, MHM, SA, J-FB: contributed to interpretation of findings and reviewing the first draft of manuscript and provided feedback; FMMcA, CMP: provided senior oversight, contributed to interpretation, and critically revised the manuscript for important intellectual content; and all authors: read and approved the final manuscript.

## Data availability

To protect participant confidentiality and because of the sensitive nature of the qualitative data, the full datasets are not publicly accessible. However, anonymized data excerpts may be made available upon reasonable request to the corresponding author, subject to ethical review and approval.

## Ethics approval and consent to participate

The study was conducted in accordance with the ethical principles of the Declaration of Helsinki. Ethical approval was obtained from the Turfloop Research Ethics Committee, University of Limpopo (Ref. TREC/XXX/2025:PG). Permissions were secured from the Limpopo Provincial Department of Health, the Capricorn District Health Office, and all clinic managers. Participation was voluntary. Written informed consent was obtained from all participants before data collection, and confidentiality and the right to withdraw were assured.

**Research in Context**

**Evidence before this study**

Previous research across sub-Saharan Africa has shown that cultural beliefs, taboos, and elder influence shape maternal diets. Most studies have treated these practices as barriers rather than as relational systems rooted in moral care. There has been limited research examining how intergenerational power, gendered hierarchies, and structural poverty intersect to determine what pregnant women can actually eat, or how digital health messages fare once they enter households where elders govern the diet.

**Added value of this study**

This study shows that grandmother authority operates through moral obligation rather than coercion, framing dietary restriction as care and protection, and extending in some households to gatekeeping of the clinical encounter itself. Pregnant women navigate competing advice through quiet negotiation, deferring publicly to elders while privately concealing supplements, timing consumption, and substituting permitted foods. Structural poverty and poor infrastructure do not compete with cultural restriction but compound it: where nutrient-dense foods are unaffordable, prohibitions on them cost nothing to observe and are never tested. Digital messaging aligned with clinical advice entered households as a shared object of discussion rather than a rival authority, supporting dialog without displacing elder control.

**Implications of all the available evidence**

Improving maternal dietary practices requires engaging intergenerational power relations and structural barriers together, because neither alone explains what is eaten. Interventions should move beyond individual knowledge transfer to include grandmothers and household decision makers, design digital messages to be shown to and discussed with elders, and support counseling that works with family authority structures. Alongside this, action on food insecurity, water, and transport remains essential to make any dietary advice attainable.

## Declaration of generative AI and AI-assisted technologies in the writing process

During the preparation of this work, the author used Co-Pilot for copy editing suggestions. After using this tool/service, the author reviewed and edited the content as needed and take full responsibility for the content of the publication.

## Funding

This study was conducted as part of a collaborative initiative between the University of Limpopo (South Africa) and University College Dublin (Ireland), supported by Research Ireland and the Department of Foreign Affairs through the Sustainable Development Goals (SDG) Challenge Grant (Grant Number: 24/FIP/SDG/13402).

## Conflict of interests

The authors report no conflicts of interest.
