# Supplementary material for: Cultural Authority and Food Choices: How Intergenerational Power Shapes Maternal Diet in Rural South Africa
Source: Curr Dev Nutr. 2026 Sep 28;10(10):109542. doi: 10.1016/j.cdnut.2026.109542 (PMC13683024; doi:10.1016/j.cdnut.2026.109542)
Supplement: Supplementary file 1 [file mmc1.docx]

**Semi-structured focus group discussion and interview guides**

# **Guide 1. Pregnant women**

**Demographic information**

1. **Age**

| 18–25 |  |
| --- | --- |
| 26–35 |  |
| 36–45 |  |
| 45–55 |  |

2. **Occupation**

| Scholar/student |  |
| --- | --- |
| Employed |  |
| Unemployed |  |

3. **Level of household income**

| 0 – R10 000 |  |
| --- | --- |
| R11 000 – R20 000 |  |
| R21 000 – R30 000 |  |
| More than R30 000 |  |

4. **Level of education**

| Primary |  |
| --- | --- |
| Secondary |  |
| Tertiary |  |
| Other |  |

**Knowledge and understanding of maternal nutrition**

- What is your understanding of maternal nutrition, and why is it important during pregnancy?
- What foods do you consider essential for a healthy pregnancy?
- Where do you get information about maternal nutrition?
- Who is best to give you advice on the best nutrition for pregnancy?

**Perceptions and experiences with maternal nutrition interventions**

- What maternal nutrition programs or support services are available in your community, and have you received any nutrition advice from healthcare workers?
- How do cultural beliefs and traditions influence your food choices during pregnancy?
- What challenges do you face in accessing nutrition education and support?

**Food and nutrition equity**

- Do all pregnant women in your community have equal access to nutritious food and maternal nutrition programs? If not, what are the barriers?
- How can maternal nutrition programs be improved to better serve pregnant women?

**Use of digital health apps for maternal nutrition**

- Have you ever used a mobile health app for pregnancy-related nutrition advice? If yes, which one?
- Do you think that digital app could be helpful to you to support your nutritional choices?
- What features would be most useful in a digital maternal nutrition app?
- What challenges do you face in using mobile health apps, and would you be willing to use one for nutrition guidance?

**Dietary practices and support systems**

- What foods do you eat during pregnancy, and have you made any changes to your diet?
- What factors influence your dietary choices and ability to maintain a healthy diet?
- What role does your household or community play in supporting your nutrition?
- What kind of support or resources would help improve maternal nutrition in your community?
- Do you take nutritional supplements before, during or after pregnancy? If so which ones do you think are best?

# **Guide 2. Grandmothers/grandfathers/elders**

**Demographic information**

1. **Age**

| Below 50 |  |
| --- | --- |
| 50–59 |  |
| 60–69 |  |
| 70 and above |  |

2. **Gender**

| Male |  |
| --- | --- |
| Female |  |

3. **Relationship to the pregnant woman**

| Mother |  |
| --- | --- |
| Mother-in-law |  |
| Father |  |
| Father-in-law |  |
| Other |  |

4. **Level of education**

| Primary |  |
| --- | --- |
| Secondary |  |
| Tertiary |  |
| Other |  |

**Knowledge and understanding of maternal nutrition**

- What foods do you consider essential for a healthy pregnancy?
- What is your understanding of maternal nutrition?
- Why is maternal nutrition important during pregnancy?
- What role as a grandmother or grandfather do you play in supporting pregnant women to meet their nutritional needs?
- How do you think your cultural beliefs and traditions influence your food choices for pregnant women?
- What challenges or barriers do you face in ensuring that pregnant women meet their nutritional needs?
- What role do you think the Department of Health could play in ensuring that maternal nutritional needs are met?

# **Guide 3. Fathers**

**Demographic information**

1. **Age**

| 18–25 |  |
| --- | --- |
| 26–35 |  |
| 36–45 |  |
| 45–55 |  |

2. **Occupation**

| Scholar/student |  |
| --- | --- |
| Employed |  |
| Unemployed |  |

3. **Level of education**

| Primary |  |
| --- | --- |
| Secondary |  |
| Tertiary |  |
| Other |  |

**Knowledge and understanding of maternal nutrition**

- What foods do you consider essential for a healthy pregnancy?
- What is your understanding of maternal nutrition?
- Why is maternal nutrition important during pregnancy?
- What role as a father do you play in supporting pregnant women to meet their nutritional needs?
- How do you think your cultural beliefs and traditions influence your food choices for pregnant women?
- What challenges or barriers do you face in ensuring that pregnant women meet their nutritional needs?
- What role do you think the Department of Health could play in ensuring that maternal nutritional needs are met?

# **Guide 4. Community leaders**

**Demographic information**

1. **Age**

| 18–25 |  |
| --- | --- |
| 26–35 |  |
| 36–45 |  |
| 45–55 |  |
| Above 55 |  |

2. **Gender**

| Male |  |
| --- | --- |
| Female |  |

3. **Position in the community**

| Traditional leader |  |
| --- | --- |
| Religious leader |  |
| Community police forum |  |
| School governing body |  |
| Other |  |

4. **Level of education**

| Primary |  |
| --- | --- |
| Secondary |  |
| Tertiary |  |
| Other |  |

**Knowledge and understanding of maternal nutrition**

- What is your understanding of maternal nutrition?
- Why do you think maternal nutrition is important during pregnancy?
- What role does the community play in supporting maternal nutritional needs?
- What challenges or barriers do pregnant women face in accessing maternal nutrition and support in your community?
- What can be done to improve access to healthy local food and maternal nutrition support in your community?
- How do you think your cultural beliefs and traditions influence your food choices for pregnant women?
- As a community leader, what kind of advice or support can be provided to strengthen efforts to improve maternal nutrition?

# **Guide 5. Community health workers interview guide**

**Demographic information**

1. **Age**

| 18–25 |  |
| --- | --- |
| 26–35 |  |
| 36–45 |  |
| 45–55 |  |

2. **Gender**

| Male |  |
| --- | --- |
| Female |  |

3. **Occupation**

| Scholar/student |  |
| --- | --- |
| Employed |  |
| Unemployed |  |

4. **Years of experience as CHW**

| Less than 2 |  |
| --- | --- |
| 3–5 |  |
| 6–10 |  |
| More than 10 |  |

5. **Level of education**

| Primary |  |
| --- | --- |
| Secondary |  |
| Tertiary |  |
| Other |  |

**Section B: Knowledge, roles, and responsibilities in maternal nutrition**

- What is your understanding of maternal nutrition, and why do you think it is important during pregnancy?
- What types of foods do you typically advise pregnant women to eat for a healthy pregnancy?
- What information or key messages do you usually share with pregnant women regarding nutrition?
- What are your roles and responsibilities in supporting pregnant women with nutrition in your community?
- Are there any maternal nutrition programs or services available in your area?
- How do you help pregnant women access these services or programs?
- Do you suggest nutritional supplements and if so which ones and why?

**Section C: Community, cultural beliefs, and practices**

- How do cultural beliefs, food taboos, or traditional practices influence what pregnant women eat in your community?
- Have you experienced any difficulties discussing nutrition with pregnant women due to strong traditional or cultural beliefs? Please explain.
- How does the broader community (families, elders, local leaders) influence maternal nutrition practices?
- Where do women usually get their nutritional advice?

**Section D: Challenges, barriers, and solutions**

- What common challenges do you face in delivering maternal nutrition support in your community?
- What challenges do pregnant women face in accessing healthy food and nutrition services or education?
- How do you or your team work to overcome these challenges?

**Section E: Equity and access**

- Do all pregnant women in your community have equal access to nutritious food and maternal nutrition support?
- If not, what are the key barriers that contribute to these inequalities?
- What strategies or interventions could help close these gaps and improve equitable access?

**Section F: Use of mobile technology**

- Have you used mobile tools or apps to provide nutrition advice or support to pregnant women? If so, which ones?
- Do you think that mobile apps could be useful to advise women about the best nutrition for pregnancy?
- What mobile health features or tools would help you better support maternal nutrition?
- What challenges do you or pregnant women face when using mobile apps for nutrition guidance?

# **Guide 6. Mother mentors interview guide**

**Demographic information**

6. **Age**

| 18–25 |  |
| --- | --- |
| 26–35 |  |
| 36–45 |  |
| 45–55 |  |

7. **Gender**

| Male |  |
| --- | --- |
| Female |  |

8. **Occupation**

| Scholar/student |  |
| --- | --- |
| Employed |  |
| Unemployed |  |

9. **Years of experience as CHW**

| Less than 2 |  |
| --- | --- |
| 3–5 |  |
| 6–10 |  |
| More than 10 |  |

10. **Level of education**

| Primary |  |
| --- | --- |
| Secondary |  |
| Tertiary |  |
| Other |  |

**Section B: Knowledge, roles, and responsibilities in maternal nutrition**

- What is your understanding of maternal nutrition, and why do you think it is important during pregnancy?
- What types of foods do you typically advise pregnant women to eat for a healthy pregnancy?
- What information or key messages do you usually share with pregnant women regarding nutrition?
- What are your roles and responsibilities in supporting pregnant women with nutrition in your community?
- Are there any maternal nutrition programs or services available in your area?
- How do you help pregnant women access these services or programs?
- Do you suggest nutritional supplements and if so which ones and why?

**Section C: Community, cultural beliefs, and practices**

- How do cultural beliefs, food taboos, or traditional practices influence what pregnant women eat in your community?
- Have you experienced any difficulties discussing nutrition with pregnant women due to strong traditional or cultural beliefs? Please explain.
- How does the broader community (families, elders, local leaders) influence maternal nutrition practices?
- Where do women usually get their nutritional advice?

**Section D: Challenges, barriers, and solutions**

- What common challenges do you face in delivering maternal nutrition support in your community?
- What challenges do pregnant women face in accessing healthy food and nutrition services or education?
- How do you or your team work to overcome these challenges?

**Section E: Equity and access**

- Do all pregnant women in your community have equal access to nutritious food and maternal nutrition support?
- If not, what are the key barriers that contribute to these inequalities?
- What strategies or interventions could help close these gaps and improve equitable access?

**Section F: Use of mobile technology**

- Have you used mobile tools or apps to provide nutrition advice or support to pregnant women? If so, which ones?
- Do you think that mobile apps could be useful to advise women about the best nutrition for pregnancy?
- What mobile health features or tools would help you better support maternal nutrition?
- What challenges do you or pregnant women face when using mobile apps for nutrition guidance?

# **Guide 7. Professional Nurse**

**Demographic information**

1. **Age**

| 18–25 |  |
| --- | --- |
| 26–35 |  |
| 36–45 |  |
| 45–55 |  |

2. **Gender**

| Male |  |
| --- | --- |
| Female |  |

3. **Occupation**

| Nurse/Midwife |  |
| --- | --- |
| Medical Officer/Doctor |  |
| Nutritionist/Dietitian |  |
| Other: Please specify |  |

4. **Years of experience**

| Less than 5 |  |
| --- | --- |
| 6–10 |  |
| 10–15 |  |
| Above 15 years |  |

5. **Primary work setting**

| Clinic |  |
| --- | --- |
| District/Referral Hospital |  |
| Antenatal Unit |  |
| Nutrition Outpatient Department |  |
| Other (Specify) |  |

**Section B: Clinical knowledge and practice in maternal nutrition**

- How would you define maternal nutrition in the context of antenatal care?
- What are the key nutrients and food groups that you emphasize for pregnant women during consultations?
- What evidence-based guidelines or protocols do you follow when advising pregnant women on nutrition?
- Do you feel adequately trained to assess and address nutrition issues among pregnant women? Why or why not?
- Have you attended any CPD sessions or workshops related to maternal nutrition in the last two years?
- Do you suggest any nutritional supplements, if so which ones and why?

**Section C: Implementation of nutrition interventions**

- What maternal nutrition interventions are currently implemented in your facility (e.g., supplementation programs, dietary counselling, food parcels)?
- How are these interventions integrated into routine antenatal care services?
- How do you screen and manage women at nutritional risk (e.g., underweight, anemia, food insecurity)?
- What multidisciplinary approaches (if any) are used to address maternal nutrition (e.g., referrals to dietitians, social workers)?

**Section D: Cultural and socio-economic considerations**

- In your experience, how do cultural beliefs or taboos affect pregnant women’s food choices and adherence to nutrition advice?
- Where do women usually get their nutritional advice?
- How do socio-economic factors (e.g., income, household food insecurity, access to health services) affect maternal nutrition in your patient population?
- What strategies have you used to adapt your counselling in culturally sensitive or resource-constrained settings?

**Section E: Challenges and systemic barriers**

- What challenges do you face when providing maternal nutrition education or interventions (e.g., time constraints, lack of materials, high caseload)?
- What limitations do pregnant women experience in accessing adequate nutrition support from your facility?
- Are there any gaps in staff training or infrastructure that hinder optimal delivery of maternal nutrition care?

**Section F: Addressing food and nutrition inequities**

- Based on your experience, do all pregnant women in your facility’s catchment area have equitable access to maternal nutrition services and nutritious food?
- What policy or programmatic changes do you think could improve maternal nutrition equity in your setting?

**Section G: Digital health tools and innovations**

- Are you currently using any mobile apps or digital platforms to support nutrition counselling or monitoring in antenatal care?
- What is your view on the use of digital tools (e.g., mobile apps, SMS reminders, WhatsApp) to deliver maternal nutrition education?
- What features or content would you recommend for a digital tool aimed at improving maternal nutrition care among healthcare providers and patients?

**Section H: Support systems and capacity building**

- Do you collaborate with community health workers, NGOs, or other sectors to promote maternal nutrition?
- What support or resources (e.g., training, educational materials, job aids) would enhance your ability to deliver quality maternal nutrition care?
- What recommendations would you make to strengthen the role of healthcare providers in maternal nutrition in rural settings?

# **Guide 8. Professional Nurse**

**Demographic information**

6. **Age**

| 18–25 |  |
| --- | --- |
| 26–35 |  |
| 36–45 |  |
| 45–55 |  |

7. **Gender**

| Male |  |
| --- | --- |
| Female |  |

8. **Occupation**

| Nurse/Midwife |  |
| --- | --- |
| Medical Officer/Doctor |  |
| Nutritionist/Dietitian |  |
| Other: Please specify |  |

9. **Years of experience**

| Less than 5 |  |
| --- | --- |
| 6–10 |  |
| 10–15 |  |
| Above 15 years |  |

10. **Primary work setting**

| Clinic |  |
| --- | --- |
| District/Referral Hospital |  |
| Antenatal Unit |  |
| Nutrition Outpatient Department |  |
| Other (Specify) |  |

**Section B: Clinical knowledge and practice in maternal nutrition**

- How would you define maternal nutrition in the context of antenatal care?
- What are the key nutrients and food groups that you emphasize for pregnant women during consultations?
- What evidence-based guidelines or protocols do you follow when advising pregnant women on nutrition?
- Do you feel adequately trained to assess and address nutrition issues among pregnant women? Why or why not?
- Have you attended any CPD sessions or workshops related to maternal nutrition in the last two years?
- Do you suggest any nutritional supplements, if so which ones and why?

**Section C: Implementation of nutrition interventions**

- What maternal nutrition interventions are currently implemented in your facility (e.g., supplementation programs, dietary counselling, food parcels)?
- How are these interventions integrated into routine antenatal care services?
- How do you screen and manage women at nutritional risk (e.g., underweight, anemia, food insecurity)?
- What multidisciplinary approaches (if any) are used to address maternal nutrition (e.g., referrals to dietitians, social workers)?

**Section D: Cultural and socio-economic considerations**

- In your experience, how do cultural beliefs or taboos affect pregnant women’s food choices and adherence to nutrition advice?
- Where do women usually get their nutritional advice?
- How do socio-economic factors (e.g., income, household food insecurity, access to health services) affect maternal nutrition in your patient population?
- What strategies have you used to adapt your counselling in culturally sensitive or resource-constrained settings?

**Section E: Challenges and systemic barriers**

- What challenges do you face when providing maternal nutrition education or interventions (e.g., time constraints, lack of materials, high caseload)?
- What limitations do pregnant women experience in accessing adequate nutrition support from your facility?
- Are there any gaps in staff training or infrastructure that hinder optimal delivery of maternal nutrition care?

**Section F: Addressing food and nutrition inequities**

- Based on your experience, do all pregnant women in your facility’s catchment area have equitable access to maternal nutrition services and nutritious food?
- What policy or programmatic changes do you think could improve maternal nutrition equity in your setting?

**Section G: Digital health tools and innovations**

- Are you currently using any mobile apps or digital platforms to support nutrition counselling or monitoring in antenatal care?
- What is your view on the use of digital tools (e.g., mobile apps, SMS reminders, WhatsApp) to deliver maternal nutrition education?
- What features or content would you recommend for a digital tool aimed at improving maternal nutrition care among healthcare providers and patients?

**Section H: Support systems and capacity building**

- Do you collaborate with community health workers, NGOs, or other sectors to promote maternal nutrition?
- What support or resources (e.g., training, educational materials, job aids) would enhance your ability to deliver quality maternal nutrition care?
- What recommendations would you make to strengthen the role of healthcare providers in maternal nutrition in rural settings?
